# Atypical adenoid cystic carcinoma of the palate during pregnancy: a case report

**DOI:** 10.3389/fonc.2026.1909932

**Published:** 2026-09-16

**Authors:** Buxun Ding, Yiwen Xu, Minhai Nie, Tiejun Zhou, Zheng Ma, Yaoqiang Liu, Xuqian Liu

**Affiliations:** 1 Oral & Maxillofacial Reconstruction and Regeneration of Luzhou Key Laboratory, Southwest Medical University, Luzhou, Sichuan, China; 2 Department of Oral Basic Medicine, The Affiliated Stomatological Hospital of Southwest Medical University, Luzhou, Sichuan, China; 3 Department of Pathology, the Affiliated Hospital of Southwest Medical University, Southwest Medical University, Luzhou, Sichuan, China; 4 Department of Oral and Maxillofacial Surgery, The Affiliated Stomatological Hospital, Southwest Medical University, Luzhou, Sichuan, China; 5 Department of Oral and Maxillofacial Surgery, the Second Hospital of Hebei Medical University, Shijiazhuang, China

**Keywords:** adenoid cystic carcinoma, case report, MYB-NFIB fusion, palate, pregnancy

## Abstract

Adenoid cystic carcinoma (ACC) of the palate is a malignant salivary gland neoplasm typically characterized by perineural invasion, recurrent MYB-NFIB fusion, and an indolent clinical course. We report an atypical case of palatal ACC arising during pregnancy that exhibited rapid progression, upregulated estrogen receptor (ER) expression, and atypical histopathology. ACC most frequently involves the parotid and palatal glands and is histologically stratified into cribriform, tubular, and solid subtypes, with solid pattern carrying the poorest prognosis. The tumor is characteristically indolent yet relentless, with a marked propensity for perineural invasion, which often manifesting as pain, facial paralysis, or lingual numbness and a preference for hematogenous over lymphatic spread, with lungs as the most common site of distant metastasis. Wide local excision remains the cornerstone of treatment. The patient first presented in early pregnancy with focal microvascular congestion and pain of the palatal mucosa. Within 24 weeks, the lesion progressed to full-thickness perforation of the palatal mucosa and underlying bone. After delivery by cesarean section at 37 weeks of gestation and hospital admission, imaging revealed bilateral cervical lymph node involvement and bilateral pulmonary metastases, a trajectory that diverges sharply from the slow-growing course typical of ACC. Immunohistochemistry demonstrated small cribriform architecture with patchy or absent expression of conventional myoepithelial markers. Fluorescence *in situ* hybridization (FISH) confirmed an MYB-NFIB fusion, the hallmark molecular alteration of ACC, anchoring the diagnosis despite the unusual phenotype. Notably, ER expression was upregulated in tumor tissue, suggesting that gestational hormonal shifts may have modulated tumor progression; however, the marked nuclear atypia, high proliferative index, and early nodal and pulmonary metastasis were more consistent with intrinsic high-grade transformation of ACC. After integrating clinical, biological, molecular, and genotypic findings and excluding morphological mimics, the final diagnosis was atypical ACC of the palate. This case underscores the need for heightened clinical vigilance toward focal palatal microvascular congestion during pregnancy, which may herald an aggressive oral malignancy in this hormonally distinct population. Early recognition, supplemented by molecular confirmation (MYB-NFIB FISH) when histology is equivocal, is essential for timely intervention and improved outcomes in atypical, pregnancy-associated ACC of the oral and maxillofacial region.

## 1 Introduction

Studies have indicated that under specific physiological conditions such as pregnancy, atypical malignancies of the head and neck may be induced. Elevated estrogen and progesterone levels may promote the development and progression of ACC, potentially resulting in atypical clinical features with or without atypical pathological findings (1, 2). We report here a case of atypical ACC occurring during pregnancy. We analyze its atypical features from the perspectives of clinical presentation, tumor biological behavior, immunohistochemistry, and molecular pathology, exclude alternative diagnoses, and explore possible mechanisms underlying its atypical features. The aim is to improve risk prediction and management for pregnant patients presenting with focal microvascular congestion of the oral mucosa, to strengthen early detection and diagnosis of ACC presenting with atypical malignant features in the oral and maxillofacial region in special populations, and ultimately to improve patient outcomes.

## 2 Case report

### 2.1 Clinical findings

A 36-year-old woman of Han Chinese ethnicity at 13 weeks of gestation presented to our hospital with a 20-day history of congestion and pain of the right upper soft palatal mucosa, first noticed after eating sugarcane. She had no significant past medical history, no chronic diseases, no regular medication, and no allergies, and there was no family history of salivary gland tumors or other malignancies. She had undergone no prior surgery, radiotherapy, or chemotherapy; psychosocially, she faced financial constraints that later limited her treatment adherence (see Section 3). Clinical examination revealed a symmetric maxillofacial appearance, a mouth opening of three finger-breadths, focal microvascular congestion of the right soft palate without tenderness on palpation, and congestion of the right lateral pharyngeal wall. No enlarged cervical lymph nodes were palpable. Given that the patient was in early pregnancy, after thorough discussion she elected to defer further investigation and treatment (expectant management), and the lesion was monitored clinically. At 35 weeks of gestation, a suspicious perforating lesion appeared on the palatal mucosa. The lesion failed to heal and progressively eroded the palatal bone, leading to ulceration and perforation. At 37 weeks of gestation, the patient underwent cesarean delivery at term, and a biopsy of the palatal lesion was obtained during the same procedure. The cesarean section was performed for obstetric reasons at full term and was not related to the palatal lesion. After delivery, ancillary molecular pathology testing was performed and the patient was admitted for further evaluation and treatment (**Figure 1**).

**Figure 1:**
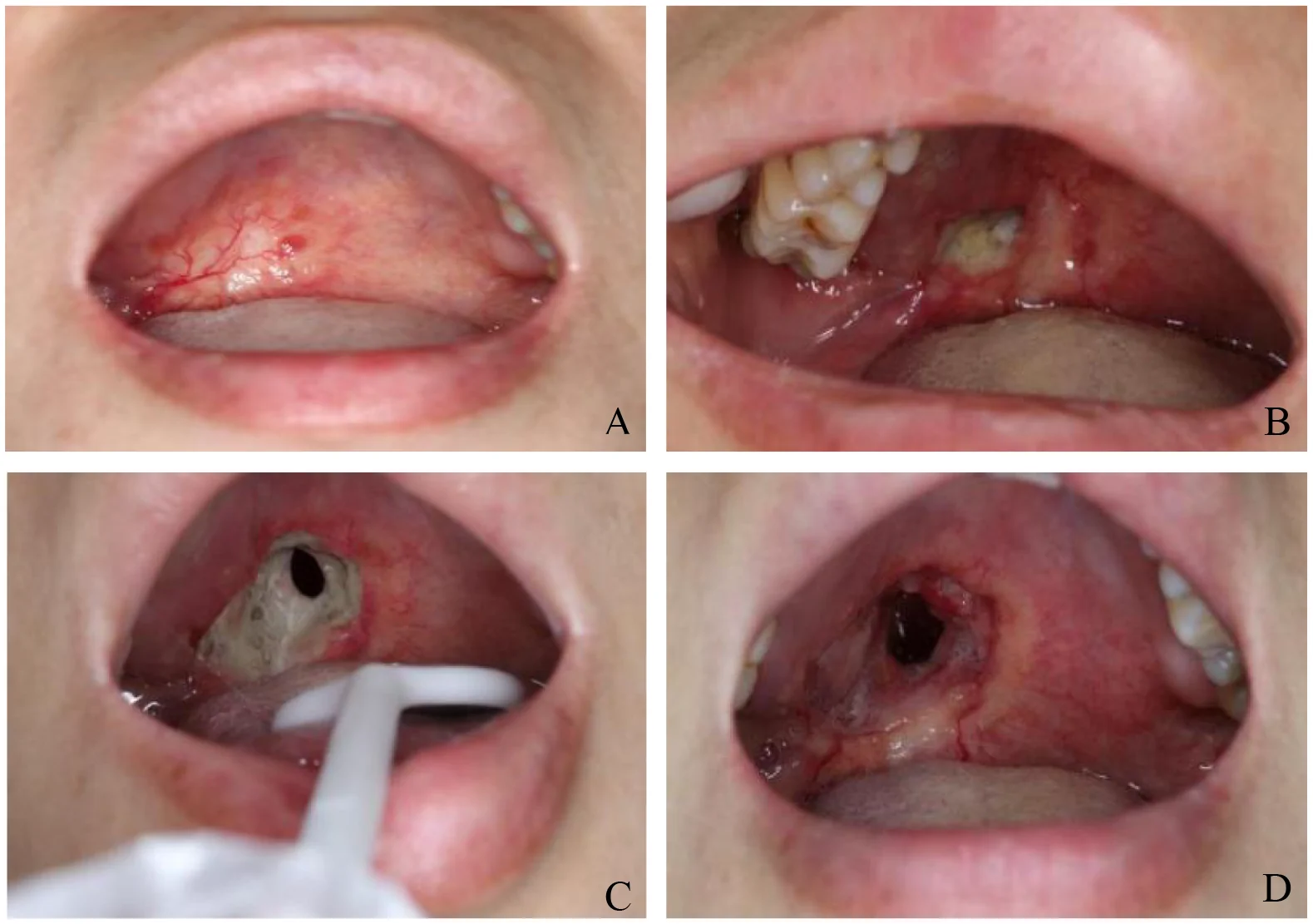
Clinical presentation: **(A)** focal microvascular congestion of the right soft palate (13 weeks’ gestation). **(B)** Mucosal perforation of the right soft palate (35 weeks’ gestation). **(C)** Mucosal and bony perforation of the right soft palate (36 weeks’ gestation). **(D)** Enlargement of the right palatal perforation (1+ week postpartum).

### 2.2 Findings

#### 2.2.1 Pathological features

Light microscopy revealed poorly differentiated tumor cells arranged in micro-cribriform structures with minimal proteoglycan deposition, marked nuclear atypia, and frequent mitotic figures. Immunohistochemistry was positive for B-cell lymphoma 2 (Bcl-2), mast/stem cell growth factor receptor (CD117), cytokeratin 7 (CK7), p53, and p63. Cytokeratin 5/6 (CK5/6), S-100 protein, and estrogen receptor (ER) showed scattered positivity. Progesterone receptor (PR), calponin, glial fibrillary acidic protein (GFAP), and cytokeratin 20 (CK20) were negative. The Ki-67 proliferation index was approximately 20%. Mucicarmine staining was negative (**Figure 2**).

**Figure 2:**
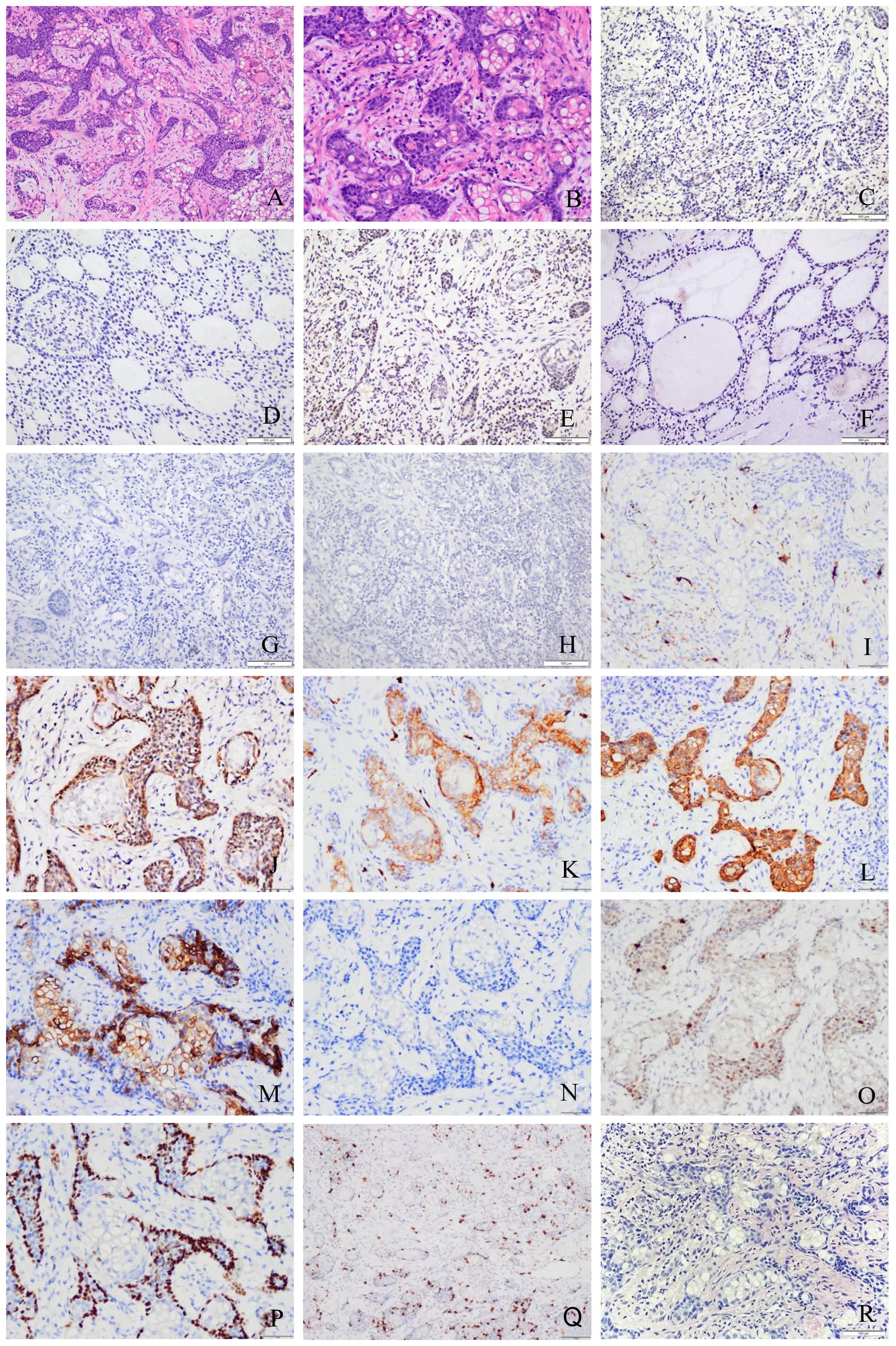
Microscopy image compilation arranged in a three-by-six grid. H&E staining [**(A)** ×100; **(B)** ×50]. **(C)** ER (±) in the present case. **(D)** ER (−) in a control ACC case. **(E)** PR (−) in the present case. **(F)** PR (−) in a control ACC case (polymer, ×200). **(G)** Calponin (−). **(H)** GFAP (−). **(I)** S-100 (±). **(J)** Bcl-2 (+). **(K)** CD117 (+). **(L)** CK5/6 (±). **(M)** CK7 (+). **(N)** CK20 (−). **(O)** p53 (+). **(P)** p63 (+) (polymer, ×200). **(Q)** Ki-67 ~20% (polymer, ×100). **(R)** Mucicarmine staining (×200).

#### 2.2.2 Imaging findings

CT showed an irregular soft-tissue density lesion on the right side of the soft palate, together with multiple solid nodules and small nodular shadows in both lungs, some with poorly defined margins. MRI demonstrated thickening of the soft palate, predominantly on the right, with perforation of the right soft palate. The imaging findings were consistent with the clinical examination findings on the right side, and no lesion was identified on the left side. In conjunction with the clinical history, malignancy was considered. Mild irregularity of the cortical bone was observed in the adjacent right upper alveolar region, and multiple lymph nodes were identified in the submental, bilateral submandibular, bilateral carotid sheath, and posterior cervical triangle regions (**Figure 3**).

**Figure 3:**
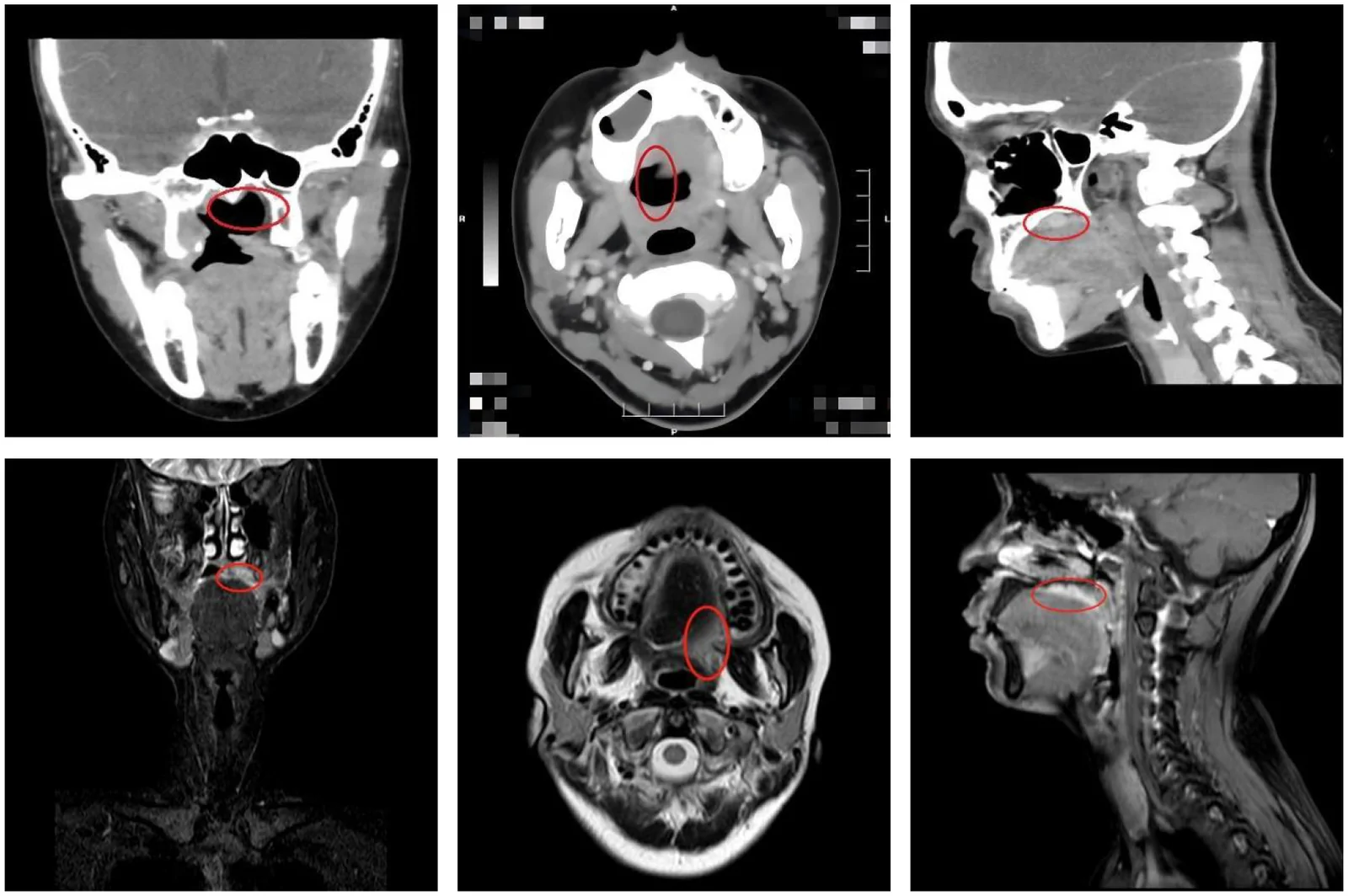
CT and MRI images in the coronal, cross-sectional, and sagittal planes of the head.

#### 2.2.3 MYB/NFIB gene fusion analysis

A dual-color MYB/NFIB break-apart probe was used to assess tumor DNA, with the orange-red (R) signal labeling MYB and the green (G) signal labeling NFIB. The normal signal pattern is 2R2G, while the typical positive (fusion) pattern is 2F1R1G (where F denotes a yellow signal produced by red–green fusion). Among 100 cells counted, the proportions were as follows: 1R1G, 7%; 2R1G, 5%; 2R2G, 11%; 1F1R, 5%; 1F1R1G, 14%; 1F2R1G, 5%; 2F, 12%; 2F1G, 7%; 2F1R, 7%; and 2F1R1G, 27%. The MYB/NFIB fusion was therefore positive (**Figure 4**).

**Figure 4:**
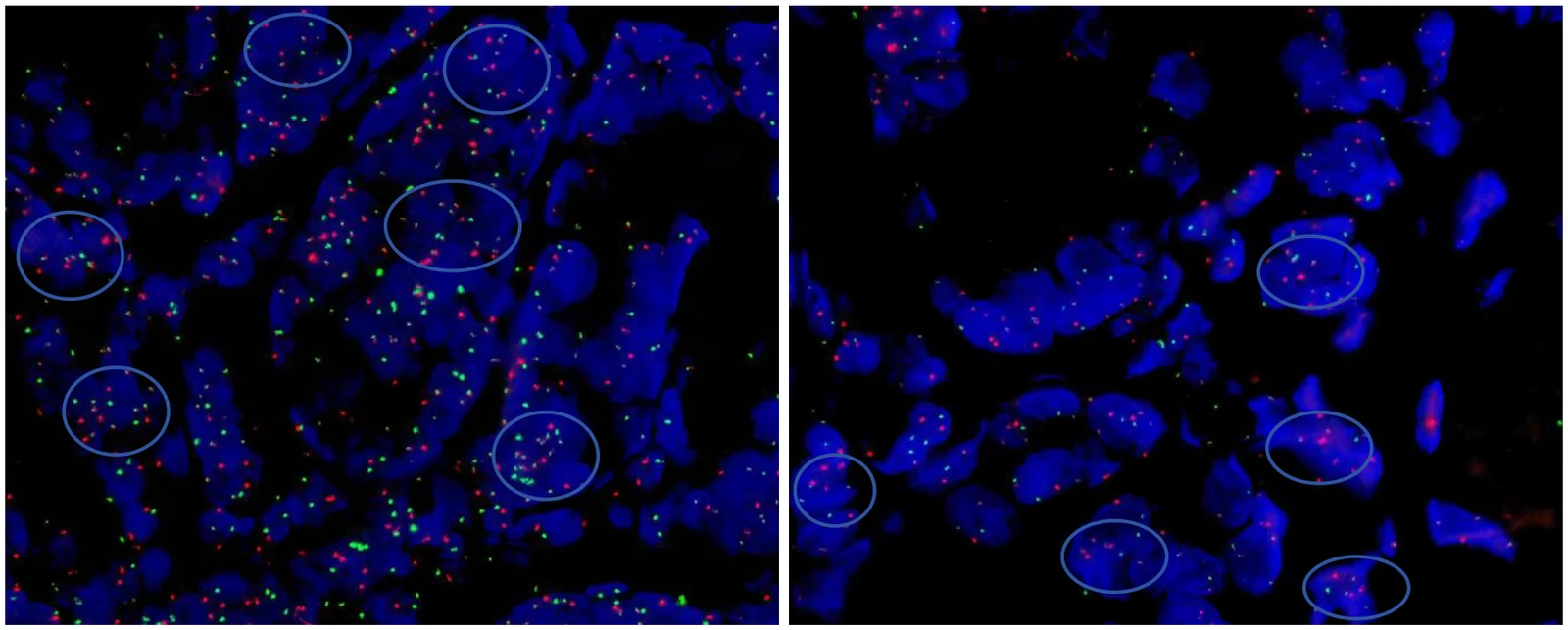
Fluorescence *in situ* hybridization.

#### 2.2.4 Diagnosis

Adenoid cystic carcinoma with bilateral cervical lymph node and bilateral pulmonary metastases.

## 3 Treatment and follow-up

After diagnosis, the patient was transferred to the Department of Oncology of the Affiliated Hospital of Southwest Medical University. Because bilateral cervical lymph node involvement and bilateral pulmonary metastases were already present at presentation, the disease was deemed surgically unresectable after multidisciplinary tumor board evaluation, and palliative systemic therapy rather than curative-intent surgery was selected. She received four cycles of the TPF regimen (docetaxel, cisplatin, and 5-fluorouracil), followed by combined anlotinib (an oral anti-angiogenic tyrosine kinase inhibitor) plus GP (gemcitabine and cisplatin) targeted therapy and chemotherapy, and was then discharged. She was advised to be readmitted for further chemotherapy in September 2022, but she declined and was lost to follow-up. A telephone follow-up was conducted in June 2024. Owing to financial difficulties, the patient had not received timely or regular treatment and was alive with disease. Mucosal perforation and palatal bone destruction had impaired her ability to eat, leading to weight loss and a poor quality of life. Informed consent was obtained from the patient and her family, and a written consent form was signed.

## 4 Discussion

ACC of the head and neck arises predominantly from secretory cells of the salivary glands, but may also originate in the lacrimal gland, nasopharynx, tongue, or external auditory canal. Its clinical manifestations depend on the primary site and the presence of perineural symptoms, with the most characteristic presentation being a slowly enlarging, firm, painless mass in the head and neck region. The lack of typical clinical features may delay medical attention and treatment.

### 4.1 Pathogenesis

The pathogenesis of ACC remains incompletely understood. Studies have shown that approximately 50% of ACCs harbor a t (6, 9) chromosomal translocation with MYB-NFIB fusion, while around 35% of ACCs lacking the t (6, 9) translocation harbor a t (8, 9) translocation with MYBL1-NFIB fusion (3, 4). Overexpression of MYB-family transcription factors may play a key role in ACC tumorigenesis (5). In our case, genotyping confirmed MYB-NFIB fusion, supporting the hypothesis that estrogen and progesterone may contribute to ACC progression and increased invasiveness.

### 4.2 Ancillary investigations

Salivary gland tumors are generally best demonstrated on MRI, which provides optimal evaluation of cranial nerve involvement and tumor infiltration around neural and bony structures. Oral ACC may erode the cortex of the mandible and infiltrate the inferior alveolar nerve, and CT can serve as a useful complementary modality. Owing to its slow growth, ACC often exhibits little uptake on ^18^F-FDG PET/CT (5). Fine-needle aspiration biopsy is the preferred initial technique for evaluating a mass, particularly in patients unsuitable for incisional biopsy. As benign and malignant tumors may share certain features, the diagnosis of ACC should integrate clinical history with ancillary investigations (6, 7). In our case, CT and MRI demonstrated soft palate perforation, multiple solid nodules and small nodular shadows in both lungs, and multiple lymph nodes in the submental, bilateral submandibular, bilateral carotid sheath, and posterior cervical triangle regions, all suggestive of malignancy. Because no overt mass was clinically apparent, fine-needle aspiration was not performed.

### 4.3 Histopathological features

ACC is classified into cribriform, tubular, and solid subtypes. The cribriform pattern is the most common, characterized by islands of basaloid cells surrounding cystic spaces of varying sizes, producing the classic “Swiss cheese” or cribriform appearance. These cystic spaces are termed pseudocysts, as they do not represent true glandular lumens and are continuous with the surrounding stroma. Pseudocysts contain basophilic glycosaminoglycans and/or eosinophilic, periodic acid–Schiff (PAS)-positive matrix material. True glandular lumina lined by cuboidal cells, displaying ductal differentiation, may also be scattered throughout the tumor; their identification can aid in diagnosis (5). In our case, light microscopy revealed poorly differentiated tumor cells arranged in microcribriform structures, with marked nuclear atypia and frequent mitoses, features that differ from those of typical (cribriform) ACC.

### 4.4 Histogenesis

The functional unit of the salivary gland is composed of luminal cells (a single layer of glandular epithelial cells and ductal luminal cells) and abluminal cells (myoepithelial and basal cells). Tumors are generally believed to arise from basal cells of the excretory ducts and from intercalated duct cells. The principal cellular components of ACC are ductal lining epithelial cells and modified myoepithelial cells, which are thought to derive from reserve cells of the intercalated ducts or from basal cells of the intercalated and excretory ducts (8). Ductal lining epithelial cells are cuboidal or ovoid, uniform in size, with round, large, deeply stained nuclei, scant clear cytoplasm, and rare mitoses. Modified myoepithelial cells appear flattened, spindle-shaped, or irregular. In the present case, the tumor cells were poorly differentiated yet relatively uniform in size, with large, round, deeply stained nuclei and scant cytoplasm, features resembling those of ductal lining cells. However, the prominent nuclear atypia, frequent mitoses, and arrangement in micro-cribriform structures with minimal proteoglycan rendered the lesion histologically atypical and precluded a definitive pathological diagnosis on morphology alone.

### 4.5 Immunohistochemistry

Salivary gland tumors can often be characterized further by immunohistochemistry. ACC is composed of altered tumor glandular epithelial and myoepithelial cells. The neoplastic glandular epithelial component typically expresses CK7, CD117, epithelial membrane antigen (EMA), CK14, CK8/18, and CAM5.2, while the neoplastic myoepithelial component expresses CK5/6, S-100, p63, p40, smooth muscle actin (SMA), and calponin (5, 8). In contrast to typical ACC, our case was calponin-negative. The Ki-67 index of approximately 20% also exceeded the ~10% characteristic of conventional ACC. PR negativity and weak ER positivity helped exclude metastatic breast carcinoma, while the negative mucicarmine stain argued against a mucinous component. Taken together, the immunophenotype and histology in this case did not conform to typical ACC.

### 4.6 Molecular pathology

Because of the cellular complexity of salivary gland tumors, definitive diagnosis can be challenging, and molecular pathology has provided a valuable adjunct. Gene sequencing and FISH have shown that approximately 90% of ACCs harbor gene rearrangements; among these, gene fusions can be detected in 95%, with MYB-NFIB fusion accounting for 60% and MYBL1-NFIB fusion for 25%, in addition to other fusions such as NFIB-TBPL1 and MYBL1-VCPIP1. The MYB-NFIB fusion is a hallmark molecular alteration in ACC and can serve as a specific diagnostic marker (9–11). FISH is rapid and can be performed on paraffin-embedded tissue. We therefore selected FISH to detect MYB-NFIB fusion in this case, which confirmed the presence of the fusion, consistent with the molecular profile of ACC. However, no single ancillary test is sufficient or confirmatory on its own: the MYB-NFIB fusion is absent in a substantial minority of ACCs, and extremely rare non-ACC tumors may harbor it. Integrating the atypical clinical features, biological behavior, immunohistochemistry, and molecular pathology, the final diagnosis was atypical adenoid cystic carcinoma. Additional confirmatory studies, such as MYB immunohistochemistry or targeted RNA sequencing, could further corroborate this diagnosis if tissue permits.

### 4.7 Differential diagnosis

The differential diagnosis of ACC includes tumors that exhibit both tubular and cribriform architecture, such as polymorphous low-grade adenocarcinoma; tumors with basaloid cytomorphology, such as basal cell adenoma, basal cell adenocarcinoma, and basaloid squamous cell carcinoma; and tumors with dual ductal and myoepithelial populations, such as pleomorphic adenoma (5, 12).

1. Polymorphous low-grade adenocarcinoma (PLGA). A common minor salivary gland malignancy, PLGA typically presents as a painless intraoral mass on the hard palate, tongue, upper lip, or tonsil, with predilection for the palate. Bleeding, telangiectasia, or mucosal ulceration may also occur. The course is prolonged, perineural invasion is common, and distant metastasis is rare (13, 14). The tumor cells are predominantly modified myoepithelial and ductal epithelial cells, often arranged in tubular, cribriform, cord-like, papillary, and cystic patterns. The cytoplasm is eosinophilic, and nuclei are round with little atypia or mitotic activity. PLGA lacks the dual ductal and myoepithelial population of ACC, and CD117 expression is typically negative or low compared with the strong CD117 expression seen in ACC. A p63 (+)/p40 (−) immunophenotype helps distinguish PLGA from other morphologically overlapping salivary gland tumors (5, 14). The marked nuclear atypia, frequent mitoses, strong CD117 expression, and rapid pulmonary metastasis seen in our case are inconsistent with PLGA, which can therefore be excluded.
2. Basal cell adenoma. A benign salivary gland tumor that occurs most commonly in the parotid gland, basal cell adenoma grows slowly, often arising near the surface of the gland, and may show a brownish appearance. It is divided into solid, trabecular, membranous, and tubular subtypes. The tumor cells exhibit a basaloid morphology and consist of two cell types: smaller cells with scant cytoplasm and round, basaloid nuclei located peripherally in the tumor nests or islands; and larger cells with abundant cytoplasm and pale nuclei located centrally (15, 16). A basement membrane–like structure surrounds the tumor nests, separating them from the surrounding connective tissue. Basal cell adenomas show β-catenin positivity on immunostaining (17, 18). In contrast to the benign nature of basal cell adenoma, our case showed Ki-67 positivity, frequent mitoses, the absence of a tumor capsule, and pain, all of which suggested malignancy.
3. Basal cell adenocarcinoma. A rare low-grade epithelial malignancy, basal cell adenocarcinoma may occasionally arise from a pre-existing basal cell adenoma. The parotid gland is the most common site, and it typically presents as a firm, often painful mass with poorly defined borders, with or without ulceration. It is composed of small basaloid cells and larger polygonal basaloid cells with eosinophilic cytoplasm, and shows moderate mitotic activity. It is classified into solid, trabecular, tubular, and membranous subtypes, with the solid subtype being most common. Basal cell adenocarcinoma shows β-catenin positivity on immunostaining (17). Both ACC and basal cell adenocarcinoma are composed of relatively uniform basaloid cells, and the solid subtype of ACC closely resembles basal cell adenocarcinoma. However, the admixture of large pale cells and small dark cells common in basal cell adenocarcinoma is not characteristic of ACC, and focal central necrosis and high mitotic activity are more frequent in ACC than in basal cell adenocarcinoma (12). In our case, no admixture of large pale and small dark cells was observed, and mitotic figures were abundant, features that are inconsistent with basal cell adenocarcinoma.
4. Pleomorphic adenoma. The most common salivary gland tumor, pleomorphic adenoma occurs most often in the parotid and palatal glands, typically presenting as a painless, slowly enlarging submucosal mass without subjective symptoms. When occurring in the palate, pleomorphic adenoma is more commonly malignant, with pain and ulceration as initial symptoms (19). It is characterized by structural pleomorphism, with epithelial and modified myoepithelial cells admixed with mucoid, myxoid, or chondroid tissue. Ductal lining cells express epithelial markers; myoepithelial cells express both epithelial and mesenchymal markers; and mucoid/chondroid components express mesenchymal markers, with S-100 and GFAP positivity (5). Our case lacked structural diversity, mucoid or chondroid tissue, and ductal architecture, and was GFAP-negative, allowing pleomorphic adenoma to be excluded.
5. Epithelial-myoepithelial carcinoma (EMC). A low-grade salivary gland malignancy with a predilection for the parotid gland, EMC usually presents as a slow-growing, painless mass. Facial nerve involvement and lymph node disease are uncommon, with frequent local recurrence but rare distant metastasis. The classic feature is a bilayered ductal structure with an inner layer of glandular epithelial cells and an outer layer of clear myoepithelial cells; the intercalated duct epithelial cells and luminal contents are mucicarmine-positive. Mitotic figures are sparse, although recurrent tumors with predominantly clear cell morphology may show prominent atypia and mitotic activity. In our case, the tumor cells exhibited prominent mitotic activity and were arranged in micro-cribriform structures without the bilayered ductal architecture typical of EMC; mucicarmine staining was negative and the spaces lacked luminal contents, allowing EMC to be excluded (20, 21).
6. Basaloid squamous cell carcinoma (BSCC). BSCC clinically resembles squamous cell carcinoma but predominantly occurs in the upper respiratory tract, and within the oral cavity it is most often seen at the base of the tongue and the floor of the mouth. The tumor consists of basaloid cells and squamous carcinoma cells, with epithelial tumor nests; multiple histological patterns may be observed, including solid lobular, cribriform, cord-like, trabecular, nested, glandular, and cystic. Nuclei show variable atypia with frequent mitoses, and necrosis may occur (22). In our case, no obvious epithelial proliferation or tumor nests were observed, and the cellular morphology differed from that of squamous carcinoma cells, allowing BSCC to be excluded.

### 4.8 Treatment and prognosis

Surgical resection is the cornerstone of ACC management, with adjuvant chemotherapy as needed. Surgery should aim for complete excision of the primary tumor; given the relatively low rate of lymph node metastasis, selective neck dissection is performed only when appropriate. Studies have demonstrated comparable rates of distant metastasis between treatment modalities, with surgery plus adjuvant radiotherapy yielding a lower recurrence rate and longer disease-free survival than surgery alone or post-recurrence radiotherapy. However, this approach has been associated with earlier distant metastasis and lower overall survival (23). Because of the high rate of distant metastasis in ACC, postoperative chemotherapy is also required. For most patients, the preferred treatment is radical surgery followed by postoperative chemotherapy, with distant metastasis being the principal determinant of outcome. At present, options for patients with metastatic disease are limited to supportive care, palliative systemic therapy, or enrollment in clinical trials, with the goal of establishing effective evidence-based treatment strategies (24). In our patient, pulmonary metastases had already developed, and after multidisciplinary evaluation she was no longer a surgical candidate. Chemotherapy was therefore selected.

### 4.9 Case summary

This was a case of ACC arising in the palate, beginning as focal microvascular congestion and pain of the palatal mucosa and progressing to perforation of the palatal bone, consistent with the recognized clinical features of ACC. The lesion did not, however, follow the typical indolent kinetics. The palatal location made early detection difficult, and the patient’s pregnancy further complicated invasive molecular pathology testing and imaging studies, all of which delayed diagnosis. As Rafizadeh et al. (25) observed in a pregnant patient with lacrimal gland ACC who also experienced rapid tumor progression, elevated estrogen and progesterone levels may contribute to ACC progression and increased invasiveness that are in line with the clinical features observed in our case.

The pathological features of this case likewise lacked the typical histology of ACC. Tumor cells were poorly differentiated and arranged in scant micro-cribriform structures with minimal proteoglycan deposition, differing from the cribriform pattern typical of ACC. Ancillary molecular pathology demonstrated p63 (+), CK5/6 (±), S-100 (±), and calponin (−), suggesting that progesterone and estrogen may have induced phenotypic changes in neoplastic myoepithelial cells, leading to loss of immunomarker expression. This is consistent with Du et al. (26), who reported that the differentiation status of neoplastic myoepithelial cells may influence both the grading and prognosis of ACC. To further investigate whether estrogen and progesterone contributed to the atypical features in this case, we evaluated ER and PR expression. The present case was ER (±) and PR (−), whereas the control case was ER (−) and PR (−). It must be emphasized, however, that serum estrogen and progesterone levels were not measured during pregnancy in this patient; the correlation between gestational hormone surges and tumor progression therefore remains speculative and hypothesis-generating.

The upregulated ER expression suggests an enhanced malignant phenotype of the tumor cells, while the uniformly negative PR indicates that PR negativity may occur in ACC irrespective of estrogen and progesterone levels.

An alternative, and in our view more compelling, explanation for the aggressive behavior of this tumor is high-grade transformation (HGT) of ACC, also termed dedifferentiated ACC which is a rare phenomenon in which a conventional ACC acquires a high-grade component characterized by loss of the dual ductal–myoepithelial population, marked nuclear atypia and mitotic activity, an elevated Ki-67 index, frequent p53 alterations, and a clinical course dominated by early lymph node and distant metastasis. Many of these features were present in our case: the tumor cells were poorly differentiated, with marked nuclear atypia and frequent mitoses; the Ki-67 index was approximately 20%, twice the level typical of conventional ACC; p53 was positive; myoepithelial differentiation was lost (calponin negativity); and the tumor progressed from a focal mucosal lesion to bony perforation with bilateral cervical nodal and bilateral pulmonary metastases within approximately 24 weeks. In our view, this case most likely represents an intrinsic high-grade transformation of ACC that has lost its slow-growing character; gestational hormonal shifts, although supported by the weak ER positivity and the clinical course, should be regarded at most as a modulating factor rather than the sole driver of tumor progression. The designation “atypical ACC” used in this report encompasses this high-grade spectrum, and we recommend that such tumors be managed with the aggressive surveillance and systemic treatment reserved for high-grade salivary gland malignancies.

These observations are in line with previous reports indicating that ACC may be ER (+)/PR (+) or ER (−)/PR (−) (27–29). Studies have shown that distant metastasis of ACC may occur as long as a decade after initial diagnosis, with many patients living with disease for several years (30, 31). In our patient, however, only 24 weeks elapsed between her initial visit at 13 weeks of gestation, when she presented with focal microvascular congestion of the right upper palatal mucosa, and her follow-up at 37 weeks, when the tumor had eroded the palatal bone, formed an ulcerative perforation, and produced multiple metastases and after multidisciplinary evaluation she was no longer a surgical candidate. Taken together, these findings indicate that this atypical ACC exhibited a more aggressive biological behavior and a higher malignant grade than usual, most plausibly reflecting intrinsic high-grade transformation, with gestational hormones possibly acting as a modulating factor.

For pregnant patients, both invasive molecular pathology testing and CT imaging, which involves radiation exposure, must be approached with caution. The difficulty of early detection of palatal ACC further hinders early diagnosis and treatment in this population. Receiver operating characteristic curve analysis showed that the ACC markers calponin, EMA, S-100, pan-cytokeratin (CK-pan), Bcl-2, CD117, p63, and p53 each provided high diagnostic accuracy (**Figure 5**). For pregnant patients, minimally invasive molecular pathology testing should be considered to enable earlier diagnosis and earlier treatment of ACC. Treatment selection should be tailored to the specific clinical situation and stage of pregnancy in order to optimize prognosis.

**Figure 5:**
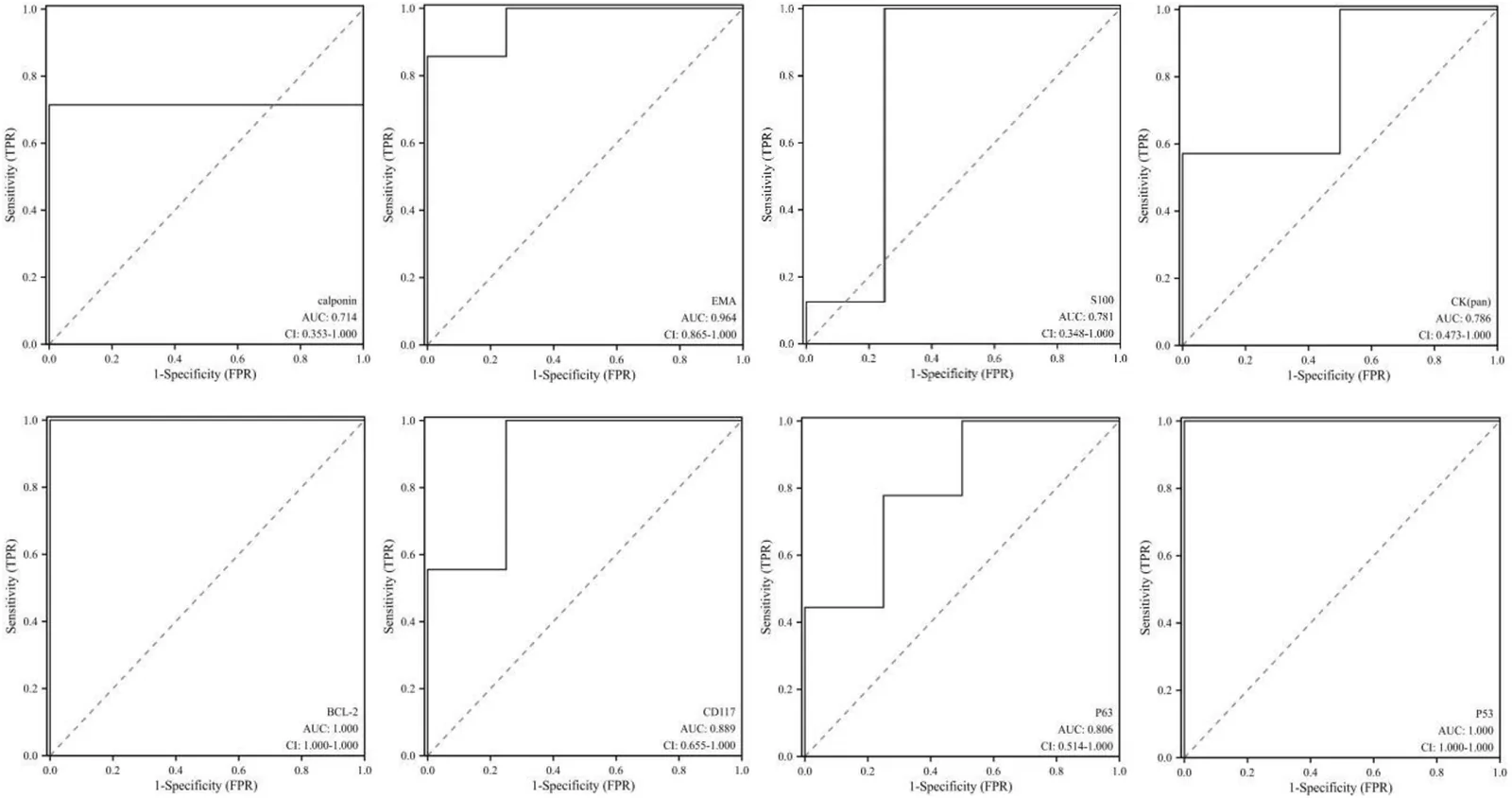
Receiver operator characteristic curve.

This case showed clinical features and biological behavior largely consistent with typical ACC but exhibited atypical pathological features, with greater malignancy and invasiveness than usual. Our aim is to alert clinicians to the possibility of malignancy heralded by rapidly progressive carcinomatous change of the oral mucosa during pregnancy, and to provide a reference for judging the optimal timing of intervention when focal microvascular congestion or mucosal destruction is observed. Because these observations rest on a single case, they should be regarded as hypothesis-generating rather than definitive, and several limitations should be acknowledged. First, causality cannot be established from a single case, and serum estrogen and progesterone levels were not measured serially during pregnancy. Second, ER expression in the tumor was weak and heterogeneous (±), so the mechanistic link between gestational hormones and the atypical phenotype remains speculative. Third, molecular confirmation relied on FISH alone; MYB immunohistochemistry and targeted RNA sequencing were not performed. Fourth, the patient declined further chemotherapy after discharge and was lost to structured follow-up for a period, and the final outcome was obtained by telephone follow-up, limiting the detail available. Finally, because the patient was not a surgical candidate, no resection specimen was available for whole-tumor evaluation. Prospective multi-institutional registries of pregnancy-associated salivary gland malignancies, paired analyses of MYB-NFIB fusion status and hormone receptor expression, and consensus protocols defining the threshold for biopsy and the sequencing of surgery, radiotherapy, and obstetric care will be required to translate insights from individual cases into evidence-based management.

## Data Availability

The raw data supporting the conclusions of this article will be made available by the authors, without undue reservation.
